# Developing Genetic Epidemiological Models to Predict Risk for Nasopharyngeal Carcinoma in High-Risk Population of China

**DOI:** 10.1371/journal.pone.0056128

**Published:** 2013-02-15

**Authors:** Hong-Lian Ruan, Hai-De Qin, Yin Yao Shugart, Jin-Xin Bei, Fu-Tian Luo, Yi-Xin Zeng, Wei-Hua Jia

**Affiliations:** 1 State Key Laboratory of Oncology in Southern China, Guangzhou, China; 2 School of Public Health, Sun Yat-Sen University, Guangzhou, China; 3 Unit of Statistical Genomics, Division of Intramural Research Program, National Institute of Mental Health (NIMH)/National Institutes of Health (NIH), Bethesda, Maryland, United States of America; The University of Texas M. D. Anderson Cancer Center, United States of America

## Abstract

To date, the only established model for assessing risk for nasopharyngeal carcinoma (NPC) relies on the sero-status of the Epstein-Barr virus (EBV). By contrast, the risk assessment models proposed here include environmental risk factors, family history of NPC, and information on genetic variants. The models were developed using epidemiological and genetic data from a large case-control study, which included 1,387 subjects with NPC and 1,459 controls of Cantonese origin. The predictive accuracy of the models were then assessed by calculating the area under the receiver-operating characteristic curves (AUC). To compare the discriminatory improvement of models with and without genetic information, we estimated the net reclassification improvement (NRI) and integrated discrimination index (IDI). Well-established environmental risk factors for NPC include consumption of salted fish and preserved vegetables and cigarette smoking (in pack years). The environmental model alone shows modest discriminatory ability (AUC = 0.68; 95% CI: 0.66, 0.70), which is only slightly increased by the addition of data on family history of NPC (AUC = 0.70; 95% CI: 0.68, 0.72). With the addition of data on genetic variants, however, our model’s discriminatory ability rises to 0.74 (95% CI: 0.72, 0.76). The improvements in NRI and IDI also suggest the potential usefulness of considering genetic variants when screening for NPC in endemic areas. If these findings are confirmed in larger cohort and population-based case-control studies, use of the new models to analyse data from NPC-endemic areas could well lead to earlier detection of NPC.

## Introduction

In most parts of the world, *nasopharyngeal carcinoma* (NPC) occurs at an annual incidence rate of <1/100,000 [1], [2], yet in South East Asia and Southern China, it is endemic. NPC appears to be most widespread in central of Guangdong province, where the city of Sihui, for instance, shows incidence rates of 30.94/100,000 in males and 13.00/100,000 in females [3]. In addition to this strikingly localized pattern of geographic distribution, NPC is also known to cluster in families in diverse populations [4], suggesting that its etiology may involve distinct risk factors.

One such factor, which has been consistently validated, is the widespread consumption of salted fish in endemic areas [5]. A meta-analysis for preserved vegetables consumption further found that, compared with individuals who eat the least amount of preserved vegetables, those with the highest intake have approximately a two-fold increase in risk for NPC [6]. Cigarette smoking, too, has been implicated as a risk factor for NPC [7]. Although these environmental risk factors are relatively well established, however, it is not yet known whether they can be used to identify increased or reduced risk for NPC. In order to build an NPC risk prediction model based on these three known environmental predictors, we recently conducted a large case-control study in Cantonese populations. As expected, results from this study independently confirm that tobacco smoking and a childhood diet rich in salted fish and preserved vegetables are all independently associated with elevated risk for NPC [8], [9]. In order to capture inherited genetic susceptibilities as well as shared environmental and behavioral risk factors, we included data on family history of NPC in our predictive model.

Genetic association and linkage studies consistently report that NPC appears to be associated with the *HLA-A* region [10]–[12], and two *genome-wide association studies* (GWAS) recently confirmed the *HLA* region’s role in NPC in southern Chinese and Taiwanese populations [13], [14]. In the southern-Chinese GWAS, researchers have not only associated three *single-nucleotide polymorphisms* (SNPs) in the *HLA* region (rs2860580, rs2894207, and rs28421666) with elevated NPC risk, they have also identified three risk-associated loci–*TNFRSF19* on 13q12 (rs9510787, rs1572072), *MDS1-EVI1* (rs6774494) on 3q26, and *CDKN2A-CDKN2B* gene cluster on 9p21 (rs1412829)–outside the *HLA* region [14]. When analysed separately, each of these SNPs is associated with only modest effects on NPC risk and is therefore of little predictive value. We hypothesized, however, that all seven SNPs taken together could be used to create a more significant genetic score for NPC risk. Using all seven SNPs associated with the disease identified in the southern Chinese GWAS, therefore, we built our first predictive genetic model for determining NPC risk.

Similar models have been successfully used to assess the risk of developing other cancers, such as the web-based risk prediction tools for lung cancer (http://nomograms.mskcc.org/Lung/RiskAssessment.aspx) and breast cancer (http://www.cancer.gov/bcrisktool/). To project individualized risk for the disease, these tools only require users to answer a few simple questions on the website. As multiple risk-carrying variants have been identified from GWAS data for such common disorders as cardiovascular diseases [15], breast cancer [16], [17], prostate cancer [18]–[20], and diabetes [21], investigators have tested whether they might increase these disease models’ predictive ability. With the growing usefulness of *Genetic Risk Prediction Studies* (GRIPS), moreover, guidelines were also developed to ensure the transparency, quality, and completeness of reported results [22].

Experience has shown that early screening and improved radiotherapy techniques can dramatically improve rates of survival from NPC. But while the five-year survival rates for early-stage (I/II) patients is 83–93%, the rate for patients diagnosed with late-stage (III/IV) NPC is only 63–72% [23]. These figures underscore the need to develop an efficient strategy and screening program for the early detection of NPC in high-risk areas.

In an attempt to meet that need, this study introduces several NPC prediction models that are the first to take into account known environmental risk factors, family history of the disease, and a genetic risk score comprising seven high risk SNPs identified from the southern Chinese GWAS. In this pilot study, we evaluate whether or not these complex models, based on a variety of risk factors, are in fact more powerful for the early detection of NPC. We propose to conduct further validation studies in future using data from our on-going large cohort study [24] and from a population-based case-control study (http://www.npcgee.com/en/index.aspx). These validation studies are a necessary step toward development of better risk prediction models for use as screening tools in areas where NPC is endemic.

## Materials and Methods

### Subjects

Subjects for this study were selected from those taking part in a large case-control study and are fully described elsewhere [8]. This study was reviewed and approved by the Human Ethics Approval Committee of *Sun Yat-Sen University Cancer Center* (SYSUCC). All patients signed informed consent before data collection.

Briefly, for the patient cohort, NPC cases were identified from the medical records of the SYSUCC in Guangzhou, the capital of Guangdong Province. Patients were histologically confirmed and enrolled in the hospital at some time between October 2005 and October 2007. All patients had had no previous diagnosis of or treatment for NPC, were without any prior history of cancer, younger than age 80, were born and continuously lived in Guangdong province at least for 5 years. Subjects with immunological and mental disease were excluded. Using these criteria, a total of 1,387 NPC cases were included in the study and 61 were excluded. Therefore, the consent rate for the NPC cases were 95.8%.

At the same time, for controls, visitors seeking physical examinations at community hospitals in 21 municipalities in Guangdong Province were interviewed. In-person interviews were completed for 1,459 (66.0%) eligible controls, who were frequency-matched to cases by age (± five years), gender, educational level, dialect, and household type (rural or urban). Controls also met the same inclusion criteria as the cases.

### Data Collection

Trained interviewers conducted live interviews, which included an extensive questionnaire, with patients in hospitals and controls in physical examination centers. Information collected included: demographic characteristics (age, sex, ethnicity, dialect, educational level, and household type), family history of NPC, and dietary and cigarette smoking habits (using data collection techniques describe in detail elsewhere) [8], [9]. Briefly, for such known NPC risk factors as salted fish and preserved vegetables, subjects were asked to choose from three categories of intake frequency: less than monthly, monthly, and weekly or more. For cigarette smoking, subjects were asked to choose from the following categories: age when began smoking, cumulative years of smoking, and type of smoking. A final statistic defining the cumulative impact of smoking in “pack-years” was determined by multiplying the number of packs of cigarettes smoked per day by the number of years the subject smoked. Meanwhile, approximately 6–7 ml of venous blood was collected. Genomic DNAs were isolated from whole blood samples using a commercial DNA extraction kit (Qiagen). Genotyping was conducted using Human610-Quad BeadChips (Illumina). Samples with a SNP call rate of <96% were removed. SNPs were excluded if they had a call rate <95%, a minor allele frequency <3% or significant deviation from Hardy-Weinberg Equilibrium in the controls (P<10^−6^) [14].

### Statistical Analysis

We used logistic regression analysis to obtain estimated odds ratios (ORs) and 95% confidence intervals (CIs) for associations between risk factors and disease. Linear trend tests were conducted on all ordinal variables. Seven SNPs with statistically significant associations with NPC in the southern Chinese GWAS [14] were selected as genetic variables for our predictive model. Although we also found minor alleles of six SNPs associated with decreased NPC risk, however, we elected to use ORs of the high-risk alleles rather than these low-frequency alleles in our comparisons across SNPs.

For simplicity’s sake, and to facilitate application of our risk model in future, we created a ‘genetic risk score’ as a measure of the cumulative effects of multiple genetic risk variants as follows:
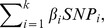
where *k* is the number of SNPs replicated in this study; SNP_i_ is the number of risk alleles (0, 1, or 2); *β_i_* is the regression coefficient for SNP_i_, which was derived using a logistic regression model.

In designing a statistical tool for NPC prediction, we constructed five models considering different mixes of factors associated with the disease: environmental risks, family history of NPC, epidemiological risks (environmental predictors and family history of NPC), genetic risks (using our genetic risk score), and an inclusive model in which all of the above were considered. A nonparametric approach was used to compare the *area under* the *receiver operating characteristic* (ROC) *curves* (AUC) for these models, [25] and the Hosmer-Lemeshow test was used to assess their final calibration. Each model’s internal performance validity was evaluated using a bootstrap method involving 2,000 replications, during which the AUC was adjusted for potential over-fitting. To quantify discriminatory improvement for models with and without the genetic risk score, we also computed the *net reclassification improvement* (NRI) and *integrated discrimination index* (IDI) [26]. To decrease possible bias arising from the exclusion of subjects with incomplete information from the analysis, we imputed missing values using the multiple-imputation method (aregImpute function of the R statistic package, see www.r-project.org). Statistical analyses were performed using Stata (version 10.0) and R (version 2.14.0).

## Results

Our study sample contains 1,387 NPC cases and 1,459 healthy controls, matched for distribution in terms of age, sex, dialect, educational level, and type of household. Both patient and control groups are roughly three-quarters male. The mean age of NPC onset is about 47 years old. *(Read* *Table 1*-*Demographic Characteristics and Socioeconomic Status of the Study Populations in Reference* [*8*] *).*

**Table 1:** Association between risk of nasopharyngeal carcinoma and seven single-nucleotide polymorphisms.

		risk allele frequency		OR (95% CI)^b^						
SNP	Allele^a^	case (%)	Control(%)	heterozygote		homozygote		OR (95% CI) per risk allele^b^		*P-*trend^c^
rs6774494	A/G	68.6	64.5	1.31	1.02–1.68	1.52	1.19–1.95	1.21	1.08–1.35	8.56×10^−4^
rs2860580	G/A	74.4	61.7	2.34	1.76–3.11	3.85	2.89–5.11	1.82	1.62–2.05	1.12×10^−24^
rs2894207	A/G	89.3	82.9	1.72	1.03–2.88	2.86	1.74–4.71	1.67	1.44–1.95	9.16×10^−11^
rs28421666	A/G	89.5	85.4	1.14	0.62–2.09	1.72	0.95–3.11	1.46	1.24–1.71	4.36×10^−6^
rs1412829	A/G	92.1	88.7	1.91	0.79–4.63	2.80	1.17–6.69	1.50	1.25–1.79	4.37×10^−6^
rs1572072	C/A	75.6	73.0	1.15	0.84–1.58	1.32	0.97–1.80	1.15	1.02–1.29	0.020
rs9510787	G/A	39.9	35.4	1.17	1.00–1.38	1.48	1.19–1.86	1.21	1.09–1.34	5.00×10^−4^

a Risk allele/reference allele.

b OR = odds ratio; CI = confidence interval. OR (95% CI) for each SNP were estimated separately using a logistic regression adjusted for age, sex, educational level, dialect, and rural or urban household type.

c *P* values for trend (two-sided) were derived from Cochran- Armitage trend tests.

### Association with NPC Risk

**Figure 1:**
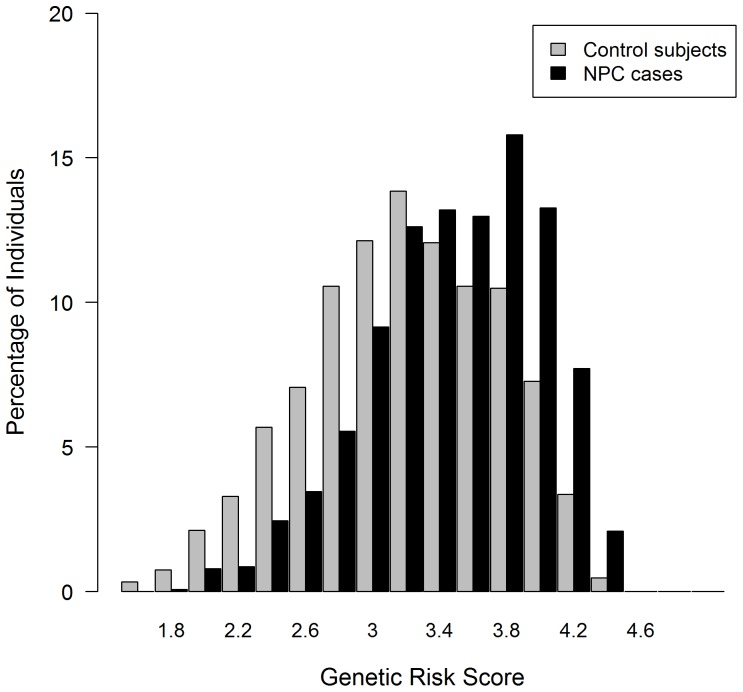
Distribution of genetic risk score. Distribution of the seven SNPs-based genetic risk score in 1,387 NPC cases (black bars) and 1,459 controls (grey bars). Individual risk for NPC was calculated by weighting each risk allele with its corresponding risk coefficient, which was derived from logistic regression.

While all seven SNPs identified in this case-control study are consistently associated with NPC risk, the most significant association occurs in SNPs: rs2860580 (OR = 1.82, 95% CI: 1.62, 2.05]), rs2894207 (OR = 1.67, 95% CI: 1.44, 1.95), and rs28421666 (OR = 1.46, 95% CI: 1.24, 1.71), located in the *HLA* region (*Table 1*). Combining risk values for all seven SNPs, we calculated a ‘genetic risk score’ for each participant. In cases, the mean (± SD) genetic risk score is 3.38±0.50, while in controls it is 3.10±0.56 (*p*<0.001). This score, moreover, is normally distributed in controls but in NPC cases is slightly skewed to the right *(* *Figure 1* *)*.

**Figure 2:**
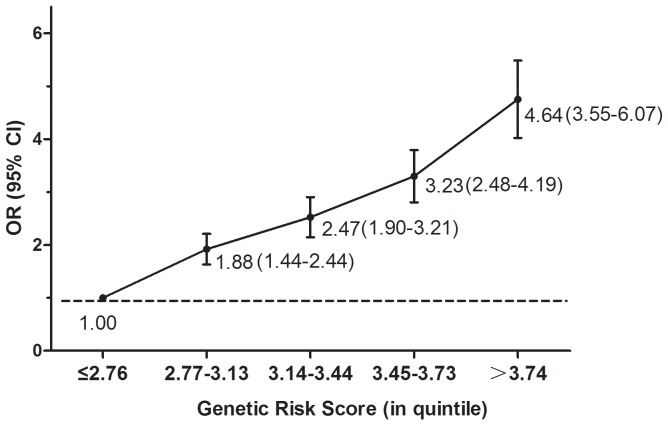
Distribution of risk for NPC by genetic risk score (in quintiles). Risk of NPC (expressed as OR ±95% CI) was adjusted for age, sex, education level, dialect, residential area, family history of NPC, pack-years smoked, salted fish and preserved vegetables consumption. The boundaries for each genetic risk score quintile are shown on the *x*-axis.

Based on the genetic risk score’s quintile distribution in subjects, we evaluated its association with NPC and other epidemiological risk factors. As expected, after adjustment for potential confounders and other epidemiological risk factors, risk for NPC rises in direct proportion to genetic risk score. Compared with people whose scores are in the lowest quintile, those with scores in the highest quintile are 4.64 times more likely to develop NPC (95% CI: 3.55, 6.07). *(* *Figure 2* *).*

Of the epidemiological factors considered, the most important risk predictors rank as follows: 1) family history of NPC (OR = 3.65, 95% CI: 2.79, 4.78); 2) weekly or more vs. less than monthly consumption of preserved vegetables (OR = 3.27, 95% CI: 2.75, 3.88) or of salted fish (OR = 2.45, 95% CI: 2.04, 2.95). We found no interaction among genetic risk score and other epidemiological factors (data not shown), and adjustment for all potentially confounding factors leaves the ORs and corresponding 95% CIs virtually unchanged. (*Table 2*).

**Table 2:** Associations between genetic variants, epidemiological risk factors and risk of nasopharyngeal carcinoma.

Predictor (code)	Case (No.)	Control (No.)	OR^a^	95% CI^a^	OR^b^	95% CI^b^
**Genetic risk score (in quintiles)**						
1 (0) = low	162	400	1.00	referent	1.00	referent
2 (1)	249	321	1.93	1.50–2.46	1.88	1.44–2.44
3 (2)	281	291	2.39	1.87–3.06	2.47	1.90–3.21
4 (3)	321	251	3.17	2.47–4.05	3.23	2.48–4.19
5 (4) = high	374	196	4.74	3.68–6.10	4.64	3.55–6.07
* P-*trend^c^			8.629×10^−38^		4.112×10^−26^	
**Family history of NPC**						
No (0)	1,154	1,382	1.00	referent	1.00	referent
Yes (1)	233	77	3.65	2.79–4.78	3.53	2.64–4.71
**Cumulative amount of smoking (in pack years)**						
≤20	955	1,110	1.00	referent	1.00	referent
>20	432	349	1.52	1.26–1.83	1.41	1.15–1.74
**Salted fish intake**						
< monthly	731	1,084	1.00	referent	1.00	referent
monthly	236	118	3.02	2.37–3.84	2.07	1.57–2.73
≥ weekly	420	257	2.45	2.04–2.95	1.55	1.25–1.92
* P-*trend^c^			1.821×10^−27^		5.767×10^−5^	
**Preserved vegetables intake**						
< monthly	555	973	1.00	referent	1.00	referent
monthly	204	129	2.81	2.20–3.58	2.07	1.56–2.74
≥ weekly	628	357	3.27	2.75–3.88	2.66	2.17–3.25
* P-*trend^c^			1.424×10^−43^		2.542×10^−12^	

a OR = odds ratio; CI = confidence interval. OR and 95% CI were derived from logistic regression, with adjustment for age, sex, education level, dialect, household type (rural/urban).

b OR and 95% CI were derived using logistic regression adjusted for age, sex, education level, dialect, rural or urban household type, and all other variables listed in the table.

c *P* values for trend (two-sided) were derived from Cochran- Armitage trend tests.

### Calibration and Classification Performance

**Figure 3:**
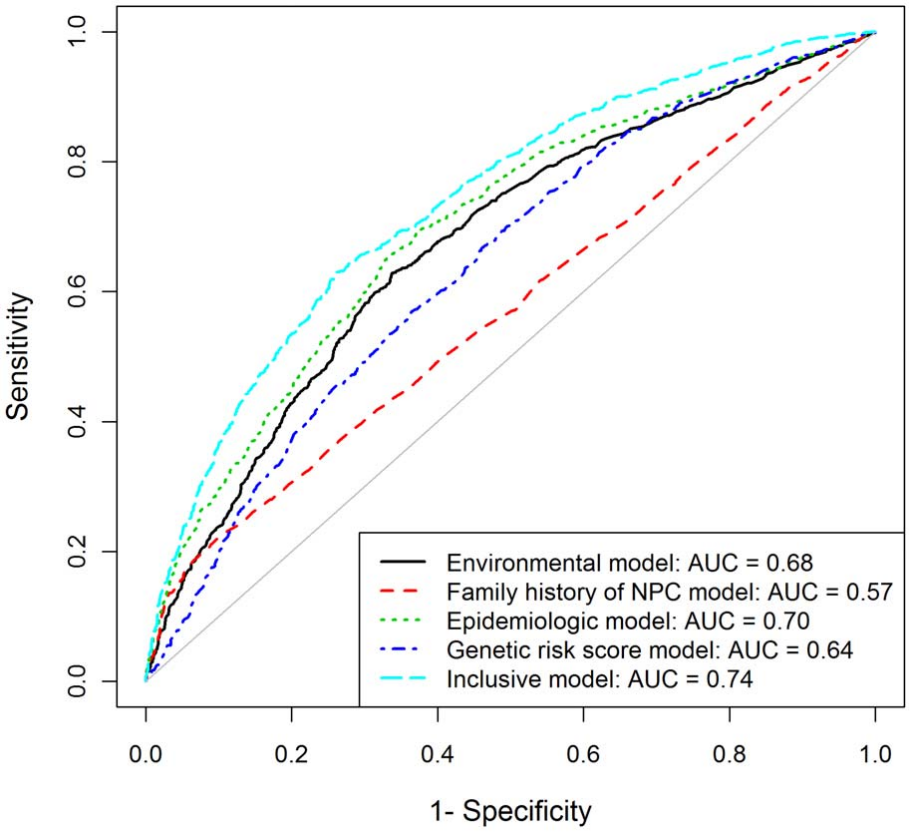
Receiver-operating characteristic (ROC) analysis. The areas under the ROC curves (AUC) as measures of predictive power for risk-assessment models based on environmental risk factors, family history of NPC, and genetic variants for NPC.

As can be seen from the following calibration statistics (Hosmer-Lemeshow χ^2^ statistic), all five of our models represent a good fit (*Table 3*). ROC curve analysis, however, shows low discriminatory accuracy for models based only on family history of NPC (AUC = 0.57), environmental predictors (AUC = 0.68), or genetic risk score (AUC = 0.64). Performance improves, however, when family history of NPC and environmental risk factors are combined (AUC = 0.70), and improves still further when genetic risk score is included (AUC = 0.74) (*Figure 3*). Statistically significant differences occur when the inclusive model is compared with the epidemiological model (difference in AUC = 0.04, *p*<0.001) or the genetic risk score model (difference in AUC = 0.10, *p*<0.001). In all models, moreover, unadjusted AUC values are slightly lower than internally validated (that is, optimism corrected) AUC values (*Table 3*). We also calculated positive predictive value (PPV) and negative predictive value (NPV) as measure of predictive ability for models based on different predictors, the two measures together with AUC consistently suggested that when more information was incorporated into the model, the discrimination ability improves accordingly (*see Table S1*).

**Table 3:** Area under curves (AUC) as a measure of predictive strength for risk-prediction models based on different indicators^a^.

Model	AUC	95% CI		AUC revisedoptimism-corrected	Model calibration		*P* value^c^
					χ^2^ statistic^b^	*P* value^b^	
Environmental	0.68		0.66–0.70	0.67	8.89	0.352	<0.001
Family history of NPC	0.57		0.55–0.59	0.55	3.53	0.897	<0.001
Epidemiological	0.70		0.68–0.72	0.69	13.01	0.112	<0.001
Genetic risk score	0.64		0.62–0.66	0.63	4.41	0.818	<0.001
Inclusive model	0.74		0.72–0.76	0.73	0.73	0.999	reference

a The environmental model is based on consumption of salted fish and preserved vegetables, and cumulative amount of smoking. The family history of NPC model includes family history of NPC only. The epidemiological model combines both environmental and family history of NPC predictors. The genetic risk score model includes a score derived from seven SNPs identified in the Cantonese GWAS. The inclusive model integrates all data on epidemiological and genetic predictors.

b χ^2^ statistic and *P* value was calculated from the Hosmer–Lemeshow Goodness-of-Fit test, a model with χ^2^ statistic <20 (*P*>0.01) is considered as a good calibration.

c AUC of the models were compared with a nonparametric approach, and *P* value was obtained from the comparison of the inclusive model with the other models.

### Reclassification for Epidemiological Model vs. Inclusive Model

To determine whether or not the inclusive model gives better classification results than the epidemiological model, we calculated both NRI and IDI. Setting the predicted risk threshold at 0.2 and 0.3, we used a reclassification table to evaluate how accurately the two models assigned people to low, intermediate, or high risk categories. In these calculations, the NRI is estimated at 0.16 and the IDI at 0.05, both of which are highly significant (*p*<0.001). (*Table 4*).

**Table 4:** Reclassification of data for use in epidemiological and inclusive models^a^.

Epidemiological Model		Inclusive Model		
Healthy controls				
	[0,0.2)	[0.2,0.3)	[0.3,1]	% reclassified
[0,0.2)	0	0	0	–
[0.2,0.3)	105	111	102	65
[0.3,1]	108	147	886	22
NPC cases				
[0,0.2)	0	0	0	–
[0.2,0.3)	12	36	74	70
[0.3,1]	28	53	1184	6
Combined Data				
[0,0.2)	0	0	0	–
[0.2,0.3)	117	147	176	67
[0.3,1]	136	200	2070	14
NRI [95% CI]: 0.16 [0.13–0.20]; *p*-value: <0.001				
IDI [95% CI]: 0.05 [0.04–0.06]; *p*-value: <0.001				

a NRI: net reclassification improvement; IDI: integrated discrimination index; Reclassification was calculated for strata of predicted risk of <0.2, 0.2 to 0.3, and ≥0.3.

To evaluate how missing data affect performance, we imputed values for missing data on education (0.35%), residence (0.77%), dialect (0.21%), family history of NPC (2.49%), pack-years smoked (1.55%), salted fish consumption (0.91%), preserved vegetables consumption (0.74%), rs6774494 (7.55%), rs2860580 (7.48%), rs2894207 (9.31%), rs28421666 (7.73%), rs1412829 (7.31%), rs1572072 (7.13%), and rs9510787 (7.41%). A secondary analysis of the data set without imputed values for missing data shows that for all five prediction models, discriminative accuracy is virtually identical to the analysis with imputation of missing data on different variables (data not shown).

## Discussion

Risk prediction models, which can evaluate the combined impact of multiple risk factors, have high potential for uncovering new insights that will improve our ability to diagnose, treat, and even prevent disease. Prediction models for breast [27] and lung [28] cancer have already been developed and validated in diverse external populations, and the Gail model is now widely used in counselling, as the basis for decisions on the use of tamoxifen for treatment, and for determining the most useful sample size in randomized prevention trials [17].

To our knowledge, however, no study has yet sought to predict NPC risk by evaluating the combined effects of its known environmental risks, family history of NPC, and genetic variants in high-risk populations. The current study incorporates all three NPC risk prediction models, validates predictability internally. With an AUC of 0.70, the resulting epidemiological model has good discriminatory ability comparable to that achieved with the Gail (AUC = 0.67) [27] and Bach (AUC = 0.72) models [28]. When a genetic risk score based on seven SNPs from the southern Chinese GWAS is incorporated into our model, moreover, the AUC increases from 0.70 to 0.74.

Since our data support earlier findings that smoking, eating preserved vegetables, and eating salted fish all elevate the risk for developing NPC significantly, we included these three variables in our environmental risk model. Other environmental exposures (drinking herbal tea or alcohol, eating inadequate fresh vegetables and fruit) were excluded to avoid mistaking spurious noise variables as independent predictors for the outcome by auto-selecting predictors (using logistic regression) from among too many variables [29]. These exclusions may, however, make our model less stable and reproducible. To achieve good performance and design a method easy enough for implementation in clinical settings, we elected to include only three predictors in our environmental models. To avoid colinearity among multiple related variables in our final model, moreover, we included only the indicator deemed most important (such as ‘cumulative amount of smoking in pack-years’ for our tobacco smoking risk factor), even though data on smoking status, age at smoking initiation, smoking intensity, duration of smoking, use of a filter beak or not, and degree of inhalation are available and significantly associated with NPC risk.

Inasmuch as family history of NPC is associated with an almost four-fold increase in NPC risk, we also explored the extent to which the family history of NPC alone, or together with other factors, helps in the identification of individuals at high risk for NPC. Since family history has, in fact, been the basis for initial risk stratification in many common and preventable conditions, it holds similar promise as the basis for a cost-effective screening tool for NPC [30], [31]. This study shows that, although family history of NPC alone has only limited predictive value, when it is incorporated into a model that also includes environmental predictors, predictive ability is substantially increased. It should also be noted that the epidemiological model estimates individual probability of developing NPC on the basis of answers to a few simple questions, making it a practical tool, following validation, for use in external populations.

As measured by the AUC, NRI, and IDI, the discrimination ability improves when common genetic variants are incorporated into the epidemiological model. These improvements in AUC and integrated discrimination rates, as well as our models’ simplicity and ease of implementation, suggest that the model might prove to be useful screening tools for NPC in endemic areas. It is worth noting that we selected only seven SNPs for inclusion in our prediction models. Of two earlier GWAS of NPC [13], [32], the GWAS in Taiwanese individuals shows a strong association at SNP rs2517713 and another independent association at rs29232 [13]. Both of these SNPs, moreover, are in considerable LD with our most significant SNP rs2860580 (rs2517713 and rs2860580: γ^2^ = 0.99, D′ = 1; rs29232 and rs2860580: γ^2^ = 0.29, D′ = 0.80). Another GWAS of NPC in a Malaysian Chinese population shows an association at *ITGA9* (on 3p21) [32], but we observed no such association and surmised that the relatively small sample size (Number of case/control = 279/512) for this earlier study may have resulted in an inconsistent observation. Taking all of the evidence together, we decided to include seven SNPs for this study. We realize, however, that this model is only preliminary and should be revised to include new independent loci as they are found. Additionally, we compared the allele frequencies of the seven SNPs in our case/control subjects with other ethnic populations. We used the data from dbSNP132 (URL: http://www.ncbi.nlm.nih.gov/snp), in which the allele frequencies were estimated in multiple ethnic groups by multiple-center human genome projects, including Japanese in Tokyo, Han Chinese in Beijing, European and Sub-Saharan African populations from the HapMap project, and multiple populations from the 1000 Genome Projects. We observed that rs9510787-G allele has a higher frequency in Cantonese compared with other ethnic groups, while rs6774494-G allele has a lower frequency compared with other ethnic groups. The allele frequencies of other five SNPs are various across different ethnic groups (*see Table S2*).

Currently, no independent large-scale case-control study has been conducted to evaluate the effect-sizes of these seven SNPs on NPC risk among other ethnic populations. Therefore, we are not able to compare the risk effect of genetic risk score across different ethnic populations. However, it is reasonable that our risk models based on epidemiological risk factors and genetic risk score might need to be carefully refined when trying to apply to other ethnic populations because of different effect-sizes of risk factors for different ethnic populations.

Modelling NPC risk using genetic risk score (susceptibility loci) and well-established risk factors for diverse ethnic populations (i.e., salted fish and preserved vegetable consumption, smoking, family history of NPC) might be of public health significance and is worthy of further investigation.

Whether genetic variants can provide estimates stable enough to be translated into disease prediction on an individual level remains to be seen [33]. In evaluating the performance of breast-cancer risk models, Sholom *et al*. consider 10 significant common genetic variants. Adding these genetic information to existing risk models, they found, only increases their AUC from 58.0% to 61.8%. This finding indicates that risk analysis based on common variants is not yet able to identify reduced or elevated individual risk in a clinically useful way [17]–a finding echoed by evaluations of risk models for other common disorders [15], [19]–[21], [34]–[39].

While such findings may seem discouraging, researchers should not underestimate the potential predictive value of genetic markers. Results from at least one permutation analysis indicated that testing for multiple susceptibility genes simultaneously can give high-to-excellent discriminative accuracy [40]. Risk models for cancer prediction may be made clinically useful with the addition of information on “missing heritability”, i.e., gene-gene interaction and the contribution from causal variants. In addition, to find the best risk prediction model for NPC, other statistical models should be explored. Using various machine learning methods (such as the *support-vector machine* (SVM), *classification and regression tree* (CART), *random forest* (RF), and the neuronal network) to explore different kinds of classifiers, for instance, could help to minimize any possibility of over-fitting.

It is important to note that our risk models do not take into account a well-known risk factor for NPC, the presence of EBV antibody titers [5]. This is because while 94.7% of NPC cases in our sample test positive for EBV *virus capsid antigen*-*IgA* (VCA/IgA), only 18.2% of our control subjects are EBV positive. In view of this highly skewed distribution, we decided it was more reasonable to include variables other than EBV antibody titers in our NPC risk models. The wisdom of this choice was recently confirmed by results from our Sihui prospective EBV serological screening study [24], in which a model testing for VCA/IgA performed well for NPC prediction in the 3^rd^ year (AUC = 0.807). Over time, however, the predictive power of this model appears to weaken, until after the 8^th^ year, AUC distribution stabilizes at about 0.64. We have also conducted the analyses focusing on the subset of EBV positive populations only. The results suggested that the performance measured by AUC was slightly improved for models based on different predictors in this EBV positive subset compared with that of the full data set (data not shown). However, we would like to interpret the results with caution due to the limitation of small sample size for EBV positive controls (n = 265). It is difficult to get an accurate estimate for the model performance. We concluded that further investigation with large sample size (e.g., large-scale prospective study design) is needed for evaluating the performance of the risk model in EBV positive populations.

Since incorporating genetic variants into an epidemiological model results in higher accuracy and better performance, it may be possible to improve the performance of the EBV model by taking genetic variants into account. Testing for genetic variants has the added value of needing to be measured only once in a lifetime, whereas EBV/IgA titer status is fluctuating and must be retested over time. Using both models together should therefore increase the power for NPC risk prediction.

In sum, this study introduces a new and reasonably reliable model for the prediction of risk for NPC. Its designers hope to refine and test this new model more broadly, in order to make it feasible for future clinical use. While the results presented here are promising, they will need validation in larger samples and a variety of independent populations before the proposed models can be introduced for use in screening programs and counselling procedures. Once the models are fully tested and revised, we hope to implement an epidemiologically based software or web-based service site the public can use to evaluate their own NPC risk by answering a few questions. If a person is willing to donate a small blood sample, we will be able to refine risk estimates based on the inclusive model. If we are successful, these new tools might be used as the basis for a new strategy for the early detection of NPC in endemic areas.

## Supporting Information

Table S1 **Predictive strength for models based on different indicators.**
(DOC)

Table S2 **The distribution of minor allele frequencies of the seven SNPs in our case/control subjects and other ethnic populations.**
(DOC)
