# Supplementary material for: Developing Genetic Epidemiological Models to Predict Risk for Nasopharyngeal Carcinoma in High-Risk Population of China
Source: PLoS One. 2013 Feb 15;8(2):e56128. doi: 10.1371/journal.pone.0056128 (PMC3574061; doi:10.1371/journal.pone.0056128)
Supplement: Table S1 — Predictive strength for models based on different indicators. (DOC) [file pone.0056128.s001.doc]

**Table S1. Predictive strength for models based on different indicators**

| Model | PPV | NPV | AUC | 95% CI | |
| --- | --- | --- | --- | --- | --- |
| Environmental | 0.64 | 0.65 | 0.68 | | 0.66-0.70 |
| Family history of NPC | 0.57 | 0.55 | 0.57 | | 0.55-0.59 |
| Epidemiological | 0.65 | 0.67 | 0.70 | | 0.68-0.72 |
| Genetic risk score | 0.59 | 0.61 | 0.64 | | 0.62-0.66 |
| Inclusive model | 0.67 | 0.68 | 0.74 | | 0.72-0.76 |
