# Supplementary material for: Developing Genetic Epidemiological Models to Predict Risk for Nasopharyngeal Carcinoma in High-Risk Population of China
Source: PLoS One. 2013 Feb 15;8(2):e56128. doi: 10.1371/journal.pone.0056128 (PMC3574061; doi:10.1371/journal.pone.0056128)
Supplement: Table S2 — The distribution of minor allele frequencies of the seven SNPs in our case/control subjects and other ethnic populations. (DOC) [file pone.0056128.s002.doc]

**Table S2. The distribution of minor allele frequencies of the seven SNPs in our case/control subjects and other ethnic populations ^a^**

|  | Minor allele frequencies (Hapmap project) | | | | | | Minor allele frequencies (1000 genome project) | | |
| --- | --- | --- | --- | --- | --- | --- | --- | --- | --- |
| SNP (minor allele) | Cantonese (case subjects) | Cantonese (control subjects) | Han Chinese in Beijing | Japanese in Tokyo | European | Sub-Saharan African | CHB+JPT | CEU | YRI |
| rs9510787 (G allele) | 0.40 | 0.35 | 0.22 | 0.22 | 0.23 | 0.04 | 0.19 | 0.18 | 0.03 |
| rs6774494 (G allele) | 0.31 | 0.36 | 0.52 | 0.42 | 0.67 | 0.43 | 0.46 | 0.73 | 0.43 |
| rs2860580 (A allele) | 0.26 | 0.39 | 0.37 | 0.08 | 0.39 | 0.51 | 0.19 | 0.38 | 0.53 |
| rs2894207 (G allele) | 0.10 | 0.18 | 0.21 | 0.13 | 0.19 | 0.12 | 0.19 | 0.15 | 0.14 |
| rs28421666 (G allele) | 0.10 | 0.15 | 0.11 | 0.06 | 0.02 | 0.11 | 0.07 | --- | 0.12 |
| rs1572072(A allele) | 0.24 | 0.27 | 0.20 | 0.29 | 0.36 | 0.23 | --- | 0.40 | 0.25 |
| rs1412829 (G allele) | 0.08 | 0.11 | 0.06 | 0.09 | 0.43 | 0.00 | 0.08 | 0.42 | --- |

^a^ The Minor Allele frequencies (MAF) in Han Chinese in Beijing, Japanese in Tokyo, European, Sub-Saharan African are from the HapMap Project, and the MAF in the 1000 genome CHB+JPT, 1000 genome CEU, 1000 genome YRI populations are from the 1000 Genome Project. The MAF data is from NCBI dbSNP 132database (<http://www.ncbi.nlm.nih.gov/snp>).
